# The prevalence of cryptococcal antigenemia in newly diagnosed HIV patients in a Southwest London cohort^☆^

**DOI:** 10.1016/j.jinf.2012.09.014

**Published:** 2013-01

**Authors:** Sheel Patel, Gee Yen Shin, Ishan Wijewardana, Sasiri Rivinda Vitharana, Ian Cormack, Mark Pakianathan, Thomas S. Harrison, Tihana Bicanic

**Affiliations:** a Courtyard Clinic, Department of Genitourinary Medicine, St George's Hospital, London SW17 0QT, UK; b Department of Virology, St George's Hospital, London SW17 0QT, UK; c Department of Infection and Immunity, St George's University of London, London SW17 0RE, UK; d Croydon University Hospital, London CR7 7YE, UK; e Clinical Infection Unit, St George's Hospital, London SW19 0QT, UK

**Keywords:** Cryptococcal meningitis, Cryptococcal antigen, Cryptococcosis, Prevalence, HIV, United Kingdom

## Abstract

**Objectives:**

To determine the prevalence of cryptococcal antigenemia in a UK HIV cohort and compare baseline characteristics of patients with and without cryptococcal antigenemia.

**Methods:**

Stored sera were retrospectively tested for cryptococcal antigen (CRAG) among newly diagnosed HIV-infected persons with CD4 < 100 cells/μL, who presented to Croydon University and St George's Hospitals, London, between January 2004 and October 2010. We assessed risk factors for cryptococcal antigenemia and patient outcomes by extracting demographic and clinical information from medical records.

**Results:**

157 patients were identified with a median age of 47 and CD4 count of 26 cells/μL. 102 (65%) were of Black race and 91 (58%) of African origin. Eight patients (5%) had positive serum CRAG. 7/8 had cryptococcal meningitis (CM) as first presentation of HIV, and 1 had sub-clinical infection. 7/8 (88%) CRAG positives were of African origin compared to 84/149 (54%) of CRAG negatives (*p* = 0.14). Other baseline characteristics did not differ significantly.

**Conclusion:**

We found a 5% prevalence of cryptococcal antigenemia in newly diagnosed HIV patients with CD4 < 100 cells/μL in southwest London, the first such data for a UK HIV cohort. Cryptococcal antigenemia occurred almost exclusively in African-born individuals. We recommend a UK CRAG screening strategy targeting newly diagnosed African HIV-infected patients with CD4 < 100 cells/μL.

## Introduction

Cryptococcal meningitis (CM) is a major opportunistic infection and a leading cause of mortality in HIV-infected patients throughout the world, causing an estimated 600,000 deaths annually, particularly in resource-limited countries.^1^ Treatment remains inadequate, with 10-week mortality between 20 and 40%, even with optimal current antifungal combinations.^2^ CM usually occurs at an advanced stage of immunosuppression, with median CD4 count below 50 cells/μL in large cohorts from developed and developing countries.^3,4^

In Europe and North America, introduction of antiretroviral therapy (ART) has been associated with a decline in CM incidence. However, in resource-limited countries, where patients frequently present late with advanced disease and CD4 count below 100 cells/μL, disease burden remains high despite availability of ART.^2,5^

Exposure to *Cryptococcus neoformans* is thought to be universal. The organism is inhaled from the environment,^6^ and genotypic evidence suggests acquisition can occur many years before the development of clinical cryptococcosis in the context of immunosuppression.^7^ Cryptococcal antigenemia (presence of cryptococcal capsular polysaccharide antigen (CRAG) in blood), can precede onset of CM by weeks to months,^8^ and presents an opportunity for early intervention with pre-emptive fluconazole therapy to prevent development of CM.

In Africa, the reported prevalence of cryptococcal antigenemia in HIV patient cohorts with CD4 counts below 100 cells/μL ranges from 2 to 13%.^8–13^ In a South African ART program, a pre-ART serum CRAG test at a titre ≥1:2 had a 28% positive predictive value for development of incident CM in the first year of ART, and was an independent predictor of mortality.^9^

Compared to the cost of CM hospitalisation and treatment, CRAG screening and fluconazole treatment are cost-effective in resource-limited settings,^11,14^ with one study estimating the screen-and-treat strategy to be cost-saving above a CRAG prevalence of 3%.^11^ Routine screening of all newly diagnosed patients with CD4 < 100 cells/μL using a novel point-of-care dipstick CRAG test (www.immy.com/products/), prior to ART initiation, is currently being piloted in South Africa^15^ and Uganda [NCT01535469].

Due to lack of prevalence data for newly diagnosed HIV patients in the United Kingdom, British HIV Association (BHIVA) Opportunistic Infection guidelines^16^ recommend serum CRAG screening only in those with symptoms suggestive of cryptococcosis and CD4 count < 200 cells/μL.

We aimed to determine the prevalence of cryptococcal antigenemia in newly diagnosed HIV patients with CD4 < 100 cells/μL in an urban Southwest London population. Our HIV clinic population is multiracial and international, with a high proportion of patients originating from Sub-Saharan Africa and significant numbers presenting late with advanced HIV at diagnosis. We also sought to compare baseline characteristics of patients with and without cryptococcal antigenemia, in order to establish whether screening should be targeted at any specific groups.

## Patients and methods

This was a retrospective cohort study conducted between April and October 2011 at Croydon University (previously Mayday) Hospital and St George's Hospital in London. Newly diagnosed patients were identified from clinic and laboratory databases using the inclusion criteria: i) age ≥18 years; ii) new confirmed positive HIV serology diagnosed for the first time between January 2004 to October 2010, with stored serum or plasma available for testing; iii) CD4 count < 100 cells/μL; iv) not yet on ART at time of stored blood sample.

The study was approved by the UK National Research Ethics committee and the Research and Development Office of St George's Hospital NHS Trust. St George's Hospital Virology laboratory stores serum for 2 years and plasma (HIV viral loads) for up to 10 years. Given the use of retrospective stored samples, plus a requirement for samples to be at least 6 months old prior to testing (to allow patients to have become established on ART, such that any retrospective positive result would not impact current clinical care), the requirement for informed consent was waived.

Stored serum or plasma samples from time of initial HIV diagnosis were anonymised prior to testing. CRAG testing was performed on serum or plasma using the Cryptococcal Latex Agglutination test (Immuno-Mycologics Inc, USA), an antibody-agglutination reaction detecting the capsular polysaccharide antigen of *C. neoformans* with a specificity and sensitivity of >95%. Samples were incubated with Pronase(Roche) at 56 °C for 15 min and analysed according to manufacturers' instructions. All samples were screened undiluted and at a 1:100 dilution. Any samples with a titre of ≥1:2 were defined as positive, and serially diluted twofold to determine the CRAG titre.

Demographic and clinical data, including CD4 count at HIV diagnosis, age, sex, ethnic group, country of origin and sexual orientation, were obtained from clinic databases by clinicians independent from the laboratory researchers. For any patients with cryptococcal antigenemia detected on retrospective testing of stored serum or plasma, clinical presentation at HIV diagnosis, results of relevant investigations, antifungal treatment, time to start of ART and development of incident or relapsed CM in the first 6 months on ART were obtained from medical notes and laboratory results review. Data were analysed using GraphPad Prism v5 (GraphPad Software, USA), using the *t*-test to compare continuous variables and the Fisher's exact test for categorical variables.

## Results

One hundred fifty seven patients fulfilled the inclusion criteria, of whom 87 (55%) were male, 128 (82%) heterosexual and 26 (17%) MSM. At HIV diagnosis, median (IQR) age was 47 (39–53) years and CD4 count was 26 (11–55) cells/μL.

One hundred two (65%) patients were of Black Race, predominantly Black African (*n* = 86, 55%). Race notwithstanding, to take into account environmental exposure to *C. neoformans*, 91 (58%) were from Africa, including 3 White and 2 Asian patients, and 39 (25%) were from the UK; other regional groupings were the West Indies (*n* = 10), mainland Europe (*n* = 7), Asia (*n* = 5) and Latin America (*n* = 4).

Eight (5%) stored serum samples tested retrospectively were positive for CRAG. On case note and laboratory results review, 7 of these were patients who had presented with CM as their first manifestation of HIV, and one was deemed to have sub-clinical infection (mild headache, serum CRAG titre performed at presentation 1:2; CSF microscopy, protein and glucose normal, CRAG 1:2 and *C. neoformans* cultures negative). African-origin patients had a serum CRAG prevalence of 8% (7/91). CM was the HIV-presenting illness in 4% (7/157) of the entire cohort, and 7% (6/91) of patients from Africa.

Table 1 compares demographic and clinical data for CRAG positive and negative patients. There were no significant differences between CRAG positive and negative groups in terms of age, CD4 count or ethnicity. All but one of the CRAG positives were from Africa: 7/8 (88%), including 6 Black African heterosexuals and one White South African MSM, compared with 84/149 (54%) of CRAG negatives (*p* = 0.14).

Table 2 shows the CSF parameters and clinical course of the 8 CRAG positive patients. All were admitted to hospital (4 were transfers into St George's from local district general hospitals). The 7 patients with CM all presented with headache and were diagnosed by lumbar puncture (LP). All received a 2-week course of amphotericin B and flucytosine and were maintained on fluconazole for a median of 11 months. ART was started at a median (range) of 4 (4–32) weeks post CM diagnosis. One patient was lost to follow-up and the other 6 followed up for a median of 30 months post CM diagnosis: all were known to be alive at 6 months and 5 of 6 at 1 year (1 transferred their care at 8 months). Two of 6 experienced CM symptom recurrence compatible with immune reconstitution inflammatory syndrome (IRIS), with negative CSF *C. neoformans* cultures, at 2 and 8 months from start of ART respectively: both were re-admitted and received a course of steroids, with resolution of symptoms.

The only patient with sub-clinical infection received fluconazole prophylaxis alone (400 mg/d for 10 weeks, then 200 mg/d). This patient reported headaches in the early months of ART, but did not receive an LP, and these resolved by 12 weeks on ART. Fluconazole was stopped after 10 months and he remained asymptomatic for a further year of follow-up.

## Discussion

To our knowledge, this is the first study to assess the prevalence of cryptococcal antigenemia in a UK HIV-infected patient cohort. At two hospitals in Southwest London comprising an international population with an African heterosexual predominance, there was a 5% prevalence of cryptococcal antigenemia in newly diagnosed patients with CD4 count < 100 cells/μL. Almost all of the CRAG positive patients were African, though the statistical power of the comparison of proportion of African patients between the CRAG positive (88%) and negative (54%) groups was limited by the cohort size.

This relatively high prevalence of cryptococcal infection, on a par with some African countries, reflects our African HIV patient predominance, and may not be generalizable to all UK HIV cohorts. Our numbers may also have been augmented by a tertiary centre referral bias of complex HIV patients with advanced disease and CM for specialist Infectious Diseases inpatient care. Four of the 8 patients had been transferred to St George's from hospitals in our sector, for whom we did not have a new HIV diagnoses denominator. Excluding those transfers would result in a conservative estimate of prevalence of cryptococcal infection in newly diagnosed HIV patients with CD4 < 100 cells/μL in southwest London of 3% (4/153), and 5% (4/84) in Africans.

In our cohort, almost all the CRAG positive patients were only diagnosed with HIV at the time of presentation with CM. Late HIV diagnoses are not exclusive to resource-limited countries: in 2010, 28% of new UK HIV diagnoses had CD4 counts < 200 cells/μL^17^ and in North America in 2008, 33% of newly HIV-diagnosed patients developed AIDS within one year.^18^ By ethnicity, late presentation is highest amongst Black Africans,^17^ and the National Institute for Clinical Excellence is promoting increased HIV testing in this group.^19^ For our African CRAG positive patients with known time between arrival to the UK and new HIV diagnosis, this ranged from 5 to 16 years, suggesting opportunities for earlier HIV diagnosis and ART, which might have prevented dissemination of latent cryptococcal infection occurring at lower CD4 counts.

For those diagnosed late, the question remains whether CRAG screening at HIV diagnosis should be routinely recommended. To be effective, screening needs to be done prior to symptomatic presentation within the antigenemic window, which ranges from weeks to months.^8^ Current BHIVA guidelines^20^ recommend excluding cryptococcal infection in symptomatic patients with CD4 < 200 cells/μL but do not advocate routine screening or fluconazole prophylaxis. CM is not a reportable disease: HPA figures of new CM diagnoses in the UK between 2006 and 2011 range from 5 to 28 cases/year, suggesting significant underreporting [C Chau, Health Protection Agency HIV&STI department, personal communication]. Based on these figures and our relatively high prevalence in a London cohort, it is difficult to extrapolate to recommendations for targeted screening in the UK.

However a new, highly sensitive and specific point-of-care dipstick CRAG lateral flow assay^21^ costs just £4 per test,^22^ and the cost of fluconazole at 400 mg/d is £5/month (St George's NHS price). On the other hand, the cost of antifungal drugs alone for a 2-week course of CM treatment is £10,000 (based on a 70 kg adult, using Liposomal Amphotericin B and flucytosine as per BHIVA recommendations,^16^ St George's NHS price). Using our conservative prevalence estimate of 5% in Africans with CD4 count < 100 cells/μL, screening 100 patients would cost £400 to identify 5 CRAG positives. Following a recently proposed algorithm for asymptomatic cryptococcal antigenemia,^23^ these would require pre-emptive fluconazole therapy until CD4 count > 200 cells/μL: 12 months' treatment of 5 patients would cost approximately £300. This approach would thus be highly cost-effective (total cost £700) even if just one case of CM (£10,000) were to be prevented, notwithstanding the prevention of morbidity and mortality associated with development of CM.

In summary, the prevalence of cryptococcal antigenemia in newly diagnosed patients with CD4 < 100 cells/μL in a Southwest London HIV cohort is on a par with many resource-limited countries and was most frequent in Africans regardless of race. Late HIV presentation remains common in the UK, particularly in Black Africans. CRAG screening using new tests and fluconazole treatment is significantly less expensive than the treatment of CM. We would therefore recommend integrating CRAG screening of African HIV-infected patients with CD4 count < 100 cells/μL with national efforts to increase HIV testing in this late-presenting group who, globally as well as in this UK HIV cohort, appear to bear the largest cryptococcal meningitis disease burden.

## Conflicts of interest

All authors have no conflicts of interest to disclose.

## Source of funding

Wellcome Trust Intermediate Fellowship to T Bicanic, WT089966.

## Figures and Tables

**Table 1:** Comparison of demographic and clinical data for CRAG positive and negative groups.

	CRAG positive	CRAG negative	*p*-Value
*N*	8	149	
Sex male *n* (%)	5 (63)	82 (55)	0.73
Heterosexual, *n* (%)	6 (75)	122 (82)	0.64
Age (y), median (IQR)	38 (25–43)	42 (34–48)	0.15
CD4/μL at diagnosis, median (IQR)	34 (5–64)	26 (12–55)	0.85
Black race, *n* (%)	6 (75)	96 (64)	0.47
African origin, *n* (%)	7 (88)	84 (54)	0.14

**Table 2:** Characteristics of the CRAG positive cohort.

Patient	Age/sex	Ethnicity	Sexuality	CD4 count at HIV diagnosis (cells/μL)	Years in UK prior to HIV diagnosis	Cryptococcal infection status	Serum CRAG titre^a^	CSF CRAG titre	ART start (weeks post CM)	IRIS/Relapse (months post CM)	Total duration fluconazole (months post CM)
1	51 M	BA	Het	7	10	Subclinical	2	2	4	N	12
2	26 M	WO	MSM	38	5	Active	16	128	4	Y(2)	8
3	35 M	WB	MSM	29	UK-Born	Active	2048	4096	4	Unknown	Unknown
4	44 F	BA	Het	72	6	Active	8192	2048	11	Y(8)	41
5	26 F	BA	Het	38	16	Active	1024	10,000	32	N	10
6	43 M	BA	Het	4	10	Active	256	4096	5	N	16
7	46 F	BA	Het	81	7	Active	256	128	4	N	7
8	35 M	BA	Het	4	Unknown	Active	1024	16,000	3	N	11

CM = cryptococcal meningitis, ART = antiretroviral therapy, BA = Black African, WO = White Other, WB = White British, Het = heterosexual, MSM = men who have sex with men.

a Obtained on retrospective testing of stored serum or plasma.
